# Density of Fast Food Outlets around Educational Facilities in Riyadh, Saudi Arabia: Geospatial Analysis

**DOI:** 10.3390/ijerph18126502

**Published:** 2021-06-16

**Authors:** Alaa Ashraf AlQurashi, Dian Kusuma, Hala AlJishi, Ali AlFaiz, Abdulaziz AlSaad

**Affiliations:** 1 Applied Clinical Research Administration, King Fahad Medical City, Riyadh 11525, Saudi Arabia; aaalqurashi@kfmc.med.sa; 2 Centre for Health Economics & Policy Innovation, Imperial College Business School, London SW7 2AZ, UK; 3 Research Services Administration, King Fahad Medical City, Riyadh 11525, Saudi Arabia; Haljishi@kfmc.med.sa (H.A.); aalfaiz@kfmc.med.sa (A.A.); 4 College of Sciences, King Saud University, Riyadh 11451, Saudi Arabia; Azizalsaad95@gmail.com

**Keywords:** density, fast food, outlets, schools, Saudi Arabia

## Abstract

Background: Childhood obesity remains a public health issue globally. The latest estimate from the World Health Organization showed that over 340 million children and adolescents aged 5–19 were overweight or obese in 2016. Objective: Our study aimed to assess the density of fast food outlets around educational facilities in Riyadh, Saudi Arabia. Methods: We employed geospatial and quantitative analyses using data on fast food outlets (from surveys conducted between November 2019 and May 2020) and educational facilities in Riyadh city. Data analyses conducted using ArcMap 10.6 and Stata 15 compared the density within 500 m and 500–1000 m from the facilities. Results: We found a high density of fast food outlets around educational facilities. Nearly 80% of fast food were within twelve-minute walking or five-minute driving distances from schools, and nearly 70% of all educational facilities had at least one fast food outlet within the buffer. We also found the densities were high within both the areas closer and the areas farther away from educational facilities. In addition, the density was significantly higher around private schools compared to government schools, and the density around girls-only schools and both-gender schools was higher than that around boys-only schools. Conclusion: There is a high density of fast food outlets around educational facilities in Saudi Arabia. Effective policies are needed to help reduce potential exposure to fast food among young people in Saudi Arabia and other countries with similar settings.

## 1. Introduction

Childhood obesity remains a public health issue globally. The latest estimate from the World Health Organization showed that over 340 million children and adolescents aged 5–19 were overweight or obese in 2016 [1]. Analysis of 1975–2016 data showed that the rising trends in children’s and adolescents’ body mass index have plateaued in many high-income countries, albeit at high levels, but have accelerated in parts of Asia [2]. Similarly, Saudi Arabia, a high-income country in the Middle East, which has become increasingly westernized over the past few decades, now has one of the highest prevalence rates of obesity, including in children [3]. The latest estimates from the NCD Risk Factor Collaboration showed that obesity rates were 14.1% and 19.7% among Saudi girls and boys (5–19 years old) in 2016, increased from 5.3% and 6.1%, respectively, in 1990. The rates were comparable to the regional average of high-income Western countries (e.g., Australia, Canada, New Zealand, the United Kingdom, and the United States) at 13.3% and 16.8%, respectively [4].

Previous studies have shown that fast food availability and accessibility are significantly associated with obesity, including among children [5,6,7,8]. However, most of the evidence is from the United States. A study in Arkansas (USA) investigated the effect of the fast food environments surrounding schools on childhood body mass index (BMI). The study indicated the decreasing influence of restaurants on a child’s BMI as its distance from school increases [5]. In addition, fast food advertising inside/outside of outlets is also prevalent. A study in the United States examined 6716 fast food restaurants near schools and found that child-directed marketing inside and on the exterior of fast food restaurants is prevalent in chain restaurants [9]. A study in Sydney (Australia) examined 9151 advertisements and found that less-healthy food products were twice as likely to be advertised close to a primary school [10]. In Saudi Arabia, a study conducted in three cities (including Riyadh) with 2906 secondary school students found that obese students were significantly less active and had less favorable dietary habits compared to non-obese students (e.g., lower intake of breakfast, fruit, and milk) [11].

Moreover, the evidence on the density of fast food outlets around schools is limited. A study in Chicago (USA) used geocoded databases of restaurant and school addresses to examine locational patterns of fast food restaurants and kindergartens, primary schools, and secondary schools. It found that fast food restaurants are concentrated within a short walking distance from schools, exposing children to poor-quality food environments in their school neighborhoods. Another study investigating fast food density in New York City (USA) showed that predominantly Black areas had higher densities of fast food than predominantly White areas, and national chains were most dense in commercial areas [12]. A study in Canada examined the food availability and accessibility among adolescents beyond the home neighborhood. It found that adolescents’ exposure to food locations is greater outside of the home neighborhood, and adolescents frequently visit such locations [13]. However, these studies are only from North America, and some are not specific to educational facilities for children. Thus, our study aimed to assess the density of fast food outlets around educational facilities in Saudi Arabia, using Riyadh city as an example.

Riyadh is the capital city of Saudi Arabia with nearly eight million people, or 27% of the country’s population. In 2015, a study of among 7930 children aged 6–16 years in Riyadh city found that obesity rates were 18.0% for girls and 18.4% for boys. When compared with the WHO-based national prevalence rate of obesity reported in 2004 (≈9.3%), the obesity rate has doubled over a 10-year period. Obesity increased significantly with higher levels of socioeconomic status [14].

## 2. Methods

We conducted geospatial analysis on the density of fast food retailers around educational facilities in Riyadh city, Saudi Arabia. There were two primary datasets: fast food outlets and educational facilities. First, we collected data on all fast food outlets from November 2019 to May 2020. We only included fast food outlets from large global chains (e.g., McDonald’s, Kentucky Fried Chick (KFC), Domino’s Pizza, Pizza Hut, and Burger King) and large local chains (Herfy, Shawarmer, TomTom, Kudu, and Maestro Pizza) [15]. Before the COVID-19 pandemic, our data collectors surveyed the city area and recorded fast food outlet location, name, and brand. After COVID-19 restrictions were introduced in the country, we continued data collection using Google Maps. Both methods were also used to cross-check the data collection results for each method.

Second, educational facility data included a comprehensive list of government and private formal and informal educational facilities in the city. Formal facilities included kindergarten, elementary school, middle school, high school, and diploma/university. Informal facilities included religious (e.g., Quran) schools. Data on schools, including addresses, were obtained from Riyadh General Directorate of Education and the Ministry of Education. We used Google Sheets and geocoding add-ons to convert each facility address into geocodes (latitude and longitude) [16,17,18].

The geospatial analyses were conducted in ArcMap 10.6 using the World Topographic Map as a basemap. We employed several geospatial tools. First, we used geoprocessing/buffer tool to generate buffers of 500 m and 1 km around facility [19,20]. Distances of 500 m and 1 km correspond with walking for 6 and 12 min or driving for 2 and 4 min, respectively. Almost all households drive in Riyadh due to the very hot weather most of the year. Children usually ask for stops at fast food outlets with their drivers. Furthermore, fast food outlets commonly provide drive-through services in Riyadh. Second, we used spatial intersect and join tools to calculate the number of retailers around each facility. We represented each facility as a point on the map. Once we obtained the density data for each facility from the geographic analyses, we tested the statistical significance of the differences in densities between within 500 m and 500–1000 m using Poisson regressions in STATA 15.1 (Stata-Corp, College Station, TX, USA).

## 3. Results

Table 1 shows the characteristics of fast food outlets and educational facilities in Riyadh city. There were a total of 708 fast food outlets in our analysis, comprising 62.4% Western chains and 37.6% Saudi chain outlets. The former included large global brands, such as Domino’s Pizza (13.8%), McDonald’s (13%), KFC (10.3%), Pizza Hut (9.6%), Burger King (7.3%), Hardee’s (5.5%), and Texas Chicken, Popeyes, and Shake Shack (2.8%). The latter included large local brands, such as Herfy (21.3%), Shawarmer (8.5%), TomTom (3.4%), Kudu (2.7%), and Maestro Pizza (1.7%). In terms of educational facilities, a total of 858 schools were included in our study, comprising 48.5% government schools and 51.5% private schools. There were 33.3% girls-only schools, 30.1% boys-only schools, and 36.6% both-gender schools. Facilities that include all grades, from kindergarten to high school, made up the largest percentage of schools (41.6%); others were elementary-school-only (28%), middle-school-only (14.8%), and high-school-only (10.4%) facilities. In addition, there were 207 international schools, 54 Quran schools, and 1 special school for deaf and mute children.

**Figure 1:**
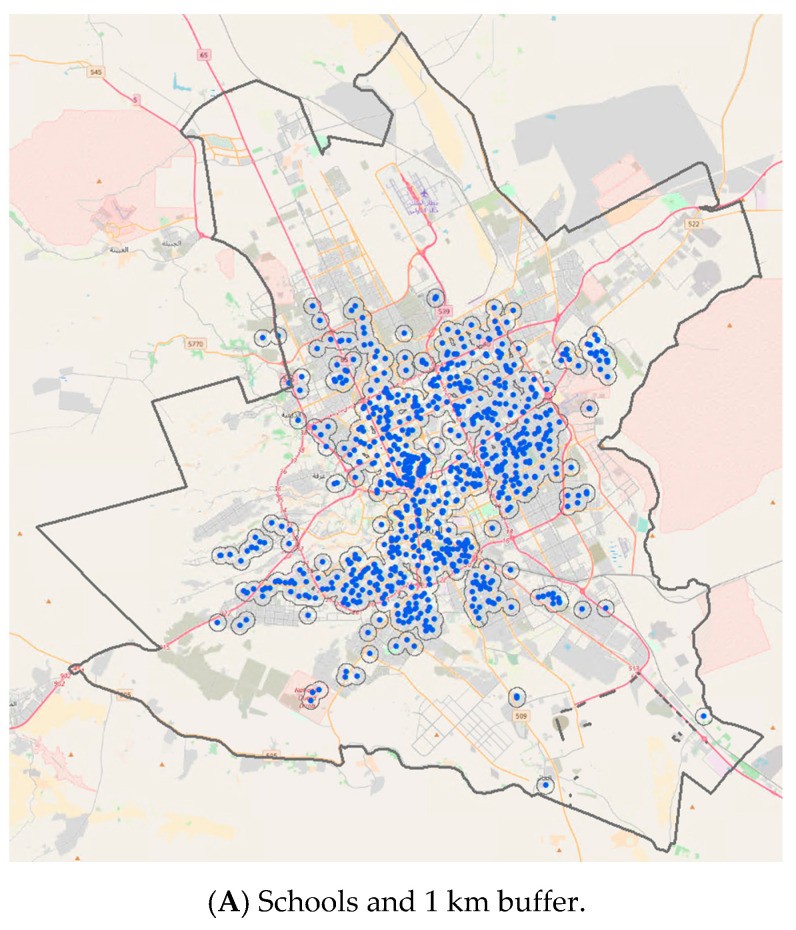

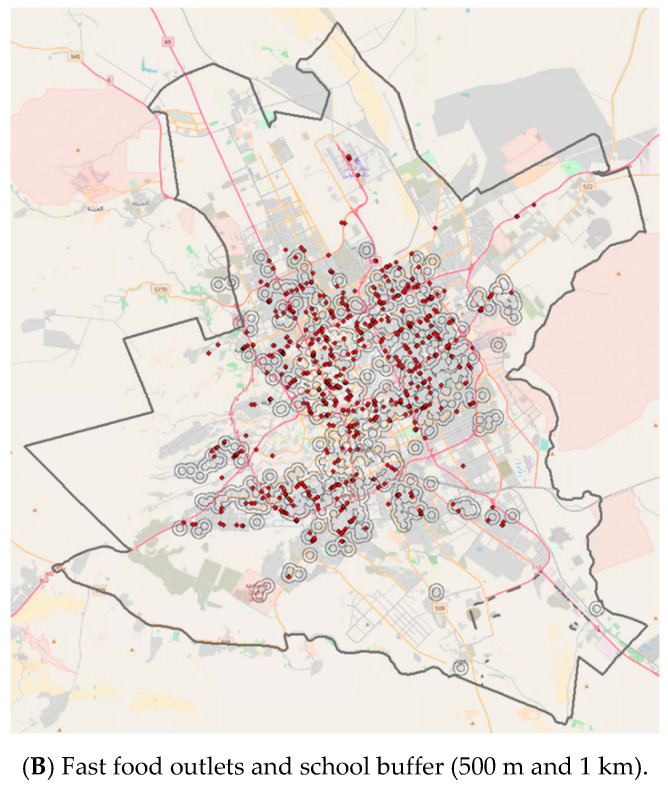
Visibility of fast food outlets around schools in Riyadh city. Note: In panel (**A**), blue dots show schools and grey lines show 1 km dissolved buffers around schools. In panel (**B**), red dots show fast food outlets and grey lines show 500 m and 1 km buffers around schools.

Figure 1 shows the maps of fast food outlets and schools in Riyadh City. In panel A, blue dots show schools and grey lines show 1 km dissolved buffers around schools. In panel B, red dots show fast food outlets and grey lines show 500 m and 1 km buffers around schools. The results show that schools and fast food outlets are distributed in all the residential and business areas of the city. Many of the outlets are within the 500 m and 1 km buffers from schools. The outer area of the city is mainly desert.

Moreover, Table 2 compares the density of fast food outlets (i.e., the number of outlets per square kilometer) in areas within 500 m of schools to that of fast food outlets within 500 m to 1 km of schools. Overall, the densities were 0.74 and 0.84 outlets per square kilometer within 500 m and 500 m–1 km, respectively, indicating a statistically significant absolute or relative difference. The significant differences were observed for government schools; girls- and boys-only schools; and elementary, middle, and high schools. For example, the densities among government schools were 0.61 and 0.69 outlets per square kilometer, while and the densities among girls-only schools were 0.74 and 0.80 outlets per square kilometer within 500 m and 500 m–1 km, respectively.

However, the density of fast food outlets within 500 m is significantly higher around private schools (0.61 per square km) than around government schools (0.86 per square km)—or 1.4 ratio for relative difference. Similarly, fast food outlets within 500 m are significantly denser around girls-only schools (0.74 per square km) and around both-gender schools (0.87 per square km) than around boys-only schools (0.59 per square km)—or a 1.3 and 1.5 ratio for relative differences, respectively.

Table 3 shows the number of fast food outlets within each school buffer (panel a) and the number of schools with at least one fast food outlet (panel b). Out of 708 fast food outlets, 42% and 79% are within 500 m and within 1 km, respectively. Furthermore, out of 858 schools, 29% and 69% have at least one fast food outlet within 500 m and within 1 km, respectively. Distances of 500 m and 1 km correspond with walking for 6 and 12 min or driving for 2 and 4 min, respectively [19,20].

## 4. Discussion

There was a high density of fast food outlets around educational facilities in Riyadh city, Saudi Arabia. We also found the density was significantly higher within 500–1000 m from schools than in areas within 500 m from schools. We found nearly 80% of those fast food outlets were within 1 km of educational facilities, which corresponds to a walking distance of under twelve minutes or a driving distance of under six minutes. Moreover, almost 70% of all educational facilities had at least one fast food outlet within the 1 km buffer. All of these findings indicate high availability and visibility of fast food outlets surrounding educational places where children and adolescents spend most of their time during the day. Moreover, these outlets are large global and local fast food chains that sell less healthy food options, such as deep-fried food (e.g., chicken and potato), potato chips, sweets, and sugar-sweetened beverage (SSB). These are all processed or ultra-processed foods, which may contribute to obesity for all age groups, including young people [21,22].

These findings also indicate potentially high exposure to advertisements for less healthy food targeted towards young people. A study of over 6700 fast food restaurants found that child-directed marketing inside and outside of fast food restaurants is prevalent in chain restaurants in the United States [9]. Another study in two cities in Indonesia found over 1500 advertisements of food and beverages, of which 32.5% and 47% were categorized as unhealthy options. The study also found that food and beverage advertisement density increased near gathering places for children and adolescents. Less healthy food and beverages were categorized using the national guidelines in regards to the sugar, salt, and fat content mentioned on the product packaging [18].

In terms of the density of fast food outlets within 500 m of educational facilities, we found that the density was higher around private schools compared to their government counterparts. This may indicate that higher income children and adolescents have potentially higher exposure to fast food outlets. Evidence from other settings show mixed results. A study examined fast food density in 5730 census block groups comprising New York City’s five boroughs to assess inequality in obesogenic environments. It found that poorer neighborhoods (e.g., predominantly Black areas) had higher densities of fast food outlets than wealthier neighborhoods (e.g., predominantly White areas). The fast food outlets were from the directory of the national chains (e.g., McDonald’s, Burger King, Kentucky Fried Chicken, Wendy’s, White Castle, and Popeye’s) and the common local ones (e.g., Crown Fried Chicken and Kennedy Fried Chicken). In those outlets, the primary menu items included hamburgers, hot dogs, and fried chicken [12]. However, another study examining 2857 census block groups in South Carolina (USA) found that, compared to areas without a fast food outlet, those with a fast food outlet had a significantly higher income and education levels. The authors asserted that this was due to spatial co-occurrence of fast food retailers around supermarket locations [4]. Compared to Saudi Arabia, fast food in the USA is more affordable for lower socioeconomic status groups and, therefore, denser around such neighborhoods. Even with no direct relationship between fast food outlet exposure and consumption observed, socioeconomic status, social norms, and income have been reported as strong contributors to the type of food consumed [23,24].

Furthermore, we found that the density of fast food outlets within 500 m of girls-only schools and both-gender schools was higher than that for boys-only schools. In term of accessibility and exposure to fast food, the high percentage of stores indicate high exposure to fast food among school aged youth for both genders. Recent evidence shows that large numbers of fast food outlets in neighborhoods are associated with higher fast food consumption, because fast food is perceived as more common and appropriate [21]. In addition, frequent snacking and consumption of fries and sweets was reported in Saudi Arabia [24,25]. It is important to understand what type of meals were eaten outside of schools. All adapted policies focused on healthy lunches and snacks at schools. Our finding could highlight the benefits of shifting the focus from the within-school environment to the outside-of-school environment.

Although our study did not measure fast food consumption using interviews or questionnaires, it is the first in Saudi Arabia to assess potential environmental factors related to poor dietary habits, especially around facilities for children and adolescents. With the increasing burden of childhood obesity in Saudi Arabia, it is crucial to use different approach such as geospatial analysis to explore associated factors related to this issue. The study also creates room for future studies to assess the type of junk food consumed by school aged children, the frequency of consumption, and social norms related to the high intake of junk food. Data from the National Health and Nutrition Examination Survey in the United States showed the caloric intake from fast food among children and adolescents between 2011 and 2012. It found that just over one-third of children and adolescents consumed fast food on a given day. In addition, children and adolescents consumed on average 12.4% of their daily calories from fast food restaurants. Caloric intake from fast foods was higher in adolescents aged 12–19 years than in children aged 2–11 years [26]. Moreover, a study examined status and risk factors for Western and Chinese fast food consumption and their associations with health outcomes among children in four mega-cities across China (e.g., Beijing, Shanghai, Nanjing, and Xi’an). The study found about half (51.9% and 43.6%) of the children consumed Western and Chinese fast food, respectively, over a period of three months. Additionally, among the children, 11.1% were obese, 19.7% were centrally obese, and 9.0% had hypertension [27].

While our study did not examine the association between the density of fast food outlets and childhood obesity rates, it should be considered in future research and policy debates. Evidence from other countries suggests that healthy public policy is an important tool for creating environments that support health and wellbeing [27]. A study identified and characterized Canadian municipalities based on their level of policy innovation and whether they adopted by-laws banning fast food drive-through services. It found that from 2002 to 2016, 27 municipalities were identified as adopters, with 22 municipalities adopting a partial ban and 5 adopting a full ban. Reasons for the drive-through bans included health promotion, environmental concerns from idling, community character and aesthetics, traffic concerns, and walkability [27]. Moreover, the city of London (UK) proposed a new planning strategy where fast food outlets should not be allowed to open within 400 m of schools. It was part of a plan to tackle childhood obesity in the city and the country. The restriction would be applied by a 400 m walking distance rather than as the crow flies [28]. A study among nearly 5500 participants in the UK examined the association between takeaway food consumption and environmental exposure to takeaway food outlets. It found that people living closest to the largest number of takeaway food outlets were more than twice as likely to be obese rather than of normal weight. The analyses were adjusted for income, as poorer people are more likely to be obese and fast food outlets are more likely to be in poorer areas [28,29].

For Saudi Arabia, this aligns with the country’s 2030 vision, which focuses on increasing public health awareness and healthy lifestyle adoption, and modifying the current infrastructure in order to make it easier for community members to adopt healthy lifestyle habits. There is an ongoing initiative by the Ministry of Health to assess the overall health of school children, including BMI. In addition, the country has recently applied extra taxation for sugary beverages and banned them from being sold at schools with the aim of controlling the contributing factors for obesity [30,31].

Our study has at least two limitations: First, our study did not measure fast food consumption or BMI among students, which limits our analysis on the link between outlet density and obesity. Second, our study only covered outlets and schools in Riyadh city, which limits the generalization to rural areas. Previous research has shown that school children consumed more fast food outside school facilities [32]. Our study opens room for further research into the type of junk food consumed, the frequency of consumption, and social norms related to fast food intake. Despite these limitations, our findings provide rigorous evidence and have important policy implications for Saudi Arabia and beyond.

## 5. Conclusions

Our aim was to assess the density of fast food outlets around educational facilities in Riyadh, Saudi Arabia. We employed geospatial and quantitative analyses using data on fast food outlets and educational facilities in the city. We found a high density of fast food outlets around educational facilities. Nearly 80% of fast food outlets were under a twelve-minute walking distance or a five-minute driving distance from schools, and nearly 70% of all educational facilities had at least one fast food outlet within the buffer. We also found the densities were high within both the areas closer and the areas farther away from educational facilities. Additionally, the density was significantly higher around private schools compared to government schools, and the density around girls-only schools and both-gender schools was higher than that around boys-only schools. Effective policies are needed to help reduce potential exposure to fast food among young people in Saudi Arabia and other countries with similar settings.

## Figures and Tables

**Table 1:** Characteristics of sample in Riyadh city.

	*n*	%
(a) Fast food outlets	708	
Western chain	442	62.4%
Saudi chain	266	37.6%
Herfy	151	21.3%
Domino’s Pizza	98	13.8%
McDonald’s	92	13.0%
KFC	73	10.3%
Pizza Hut	68	9.6%
Shawarmer	60	8.5%
Burger King	52	7.3%
Hardee’s	39	5.5%
TomTom	24	3.4%
Kudu	19	2.7%
Maestro Pizza	12	1.7%
Texas Chicken	9	1.3%
Popeyes	6	0.8%
Shake Shack	5	0.7%
(b) Schools	858	
Government	416	48.5%
Private	442	51.5%
Girls only	286	33.3%
Boys only	258	30.1%
Both	314	36.6%
All grades (kindergarten to high school)	357	41.6%
Kindergarten only	41	4.8%
Elementary school only	240	28.0%
Middle school only	127	14.8%
High school only	89	10.4%
Diploma/university	4	0.5%

Note: Saudi chains = Herfy, Kudu, Maestro Pizza, Shawarmer, and TomTom. Among 858 schools, there were 207 international schools, 54 Quran schools, and 1 special school (for deaf and mute children).

**Table 2:** Density of fast food outlets around schools in Riyadh City.

		Density per Square km				Comparison		
	*n*	<500 m		500 m–1 km		Difference	Ratio	*p*-Value
		Density	SD	Density	SD			
	[1]	[2]		[3]		[4] = [3 − 2]	[5] = [2/3]	[6]
All schools	858	0.74	1.59	0.84	1.00	0.10	1.13	<0.001
Government	416	0.61	1.53	0.69	0.91	0.07	1.12	<0.001
Private	442	0.86	1.65	0.98	1.07	0.12	1.14	0.202
Girls only	286	0.74	1.65	0.80	0.99	0.07	1.09	<0.001
Boys only	258	0.59	1.50	0.59	0.82	0.01	1.01	0.001
Both	314	0.87	1.61	1.07	1.10	0.20	1.23	0.112
All grades	357	0.86	1.65	0.98	1.06	0.12	1.15	0.372
Kindergarten only	41	0.99	1.56	1.14	1.17	0.15	1.16	0.188
Elementary school only	240	0.65	1.72	0.81	1.02	0.15	1.23	<0.001
Middle school only	127	0.51	1.12	0.54	0.70	0.03	1.06	0.001
High school only	89	0.75	1.64	0.65	0.89	−0.10	0.86	<0.001
Diploma/university	4	0.00	0.00	0.64	0.74	0.64	N/A	N/A

Note: *n* = sample, m = meter, km = kilometer, SD = standard deviation. Density is the number of schools per square kilometer. Density calculations were conducted in ArcMap 6.10. *p*-values (column 6) show the statistical significance of the difference (column 4) using Poisson regressions in STATA 15.1.

**Table 3:** Fast food outlets within buffer and schools with at least one outlet in Riyadh City.

(a) Fast food outlets within dissolved school buffer					
	**Total Outlets**	**<500 m**		**<1 km**	
	***n***	***n***	**% of Total**	***n***	**% of Total**
All schools	708	297	42%	558	79%
Government	708	153	22%	405	57%
Private	708	211	30%	449	63%
(b) Number of schools and % of total with at least one outlet within school buffer					
	**Total Schools**	**<500 m**		**<1 km**	
	***n***	***n***	**% of Total**	***n***	**% of Total**
All schools	858	245	29%	591	69%
Government	416	94	23%	252	61%
Private	442	151	34%	339	77%

Note: *n* = sample, m = meter, km = kilometer. Calculations were conducted in ArcMap 6.10.

## Data Availability

The datasets used and/or analyzed during the current study are available from the corresponding author on reasonable request.
